# Antioxidant Capability and Physical Exercise in Neurobiology: A Focus in Neurodegeneration

**DOI:** 10.3390/antiox10020250

**Published:** 2021-02-06

**Authors:** Giorgia Scarfò, Simona Daniele, Ferdinando Franzoni

**Affiliations:** 1 Department of Clinical and Experimental Medicine, University of Pisa, 56126 Pisa, Italy; giorgiascarfo91@gmail.com; 2 Department of Pharmacy, University of Pisa, 56126 Pisa, Italy; simona.daniele@unipi.it

Neurodegeneration is a complex process controlled by both genetic and environmental factors. In particular, the oxidative stress status in brain cells seems to have a crucial role in affecting negatively the onset and the progression of neurodegenerative disorders. Indeed, neuronal cells are particularly vulnerable to the oxidative damage because of their high oxygen consumption, their weak antioxidant defences, and high content of polyunsaturated fatty acids (PUFAs) in their membranes. The resulting oxidation-reduction imbalance leads to an excess of reactive oxygen and nitrogen species (RONS) that reacts with membrane PUFAs causing their peroxidation. RONS are also able to influence protein homeostasis, by causing translation errors in vivo altering protein structure, and function.

Nevertheless, several endogenous or exogenous mechanisms are able to counteract oxidative damages, such as enzymatic systems or non-enzymatic molecules acting as preventive or repair-molecules. In this regard, improving the levels of antioxidant molecules, by increasing their food consumption, could have a protective effect on neurodegeneration. This neuroprotective effect has been particularly evidenced when antioxidant molecules are associated with a regular physical activity. In fact, a regular exercise is fundamental in enhancing neuroplasticity and growth factor expression and in decreasing inflammatory states and the oxidative stress status.

This Special Issue provides an overview of the current knowledge on the relationship between the oxidative stress and neurodegenerative disorders: in particular, the aim of the present papers was to analyse brain adaptive response to a higher antioxidant consumption and regular physical exercise with special focus on the enhancement of brain antioxidant capability and the consequent improvement of cognitive functions. Lorinczova et al. [1] showed how a 6-week co-administration of iron and curcumin was able to increase brain-derived neurotrophic factor (BDNF) serum level more than iron ingestion alone in healthy adults. In terms of neuroprotection, this is fundamental since BDNF regulates brain plasticity, energy homeostasis and cognitive capabilities. In another study, Mursaleen et al. [2] considered the antioxidant N-acetylcystein (NAC) alone and associated with deferoxamine (DFO) that is an iron chelator. In particular, they formulated NAC and NAC+DFO nanocarriers targeted to mitochondria of Parkinson’s disease cellular models demonstrating a better brain availability and a more effective protection against the oxidative stress. Zyśk et al. [3] instead, assuming that N-acetyl aspartate alteration affects brain energy status, studied the impact of Acetyl-CoA and aspartate shortages in modified cholinergic neuron models. Specifically, they showed that there was a reduced N-acetyl aspartate synthesis in Zn^2+^ deregulated cellular models, with a resulting mitochondrial acetyl-CoA and aspartate shortages. As far as physical activity concerns, Cammisuli et al. [4] contributed to point out the beneficial effects of regular exercise and a healthy diet on cognitive and motor function in Parkinson’s disease patients and their possible future use as complementary strategies in neurodegenerative disorders.

## Data Availability

Not applicable
